# Biodiversity Loss from Freshwater Use for China’s Electricity Generation

**DOI:** 10.1021/acs.est.1c07155

**Published:** 2022-02-18

**Authors:** Yi Jin, Paul Behrens, Arnold Tukker, Laura Scherer

**Affiliations:** † Institute of Environmental Sciences (CML), Leiden University, 2333 CC Leiden, The Netherlands; ‡ Leiden University College The Hague, Leiden University, 2595 DG The Hague, The Netherlands; § Netherlands Organization for Applied Scientific Research (TNO), 2595 DA The Hague, The Netherlands

**Keywords:** power generation, power transmission, water consumption, thermal emissions, biodiversity impacts

## Abstract

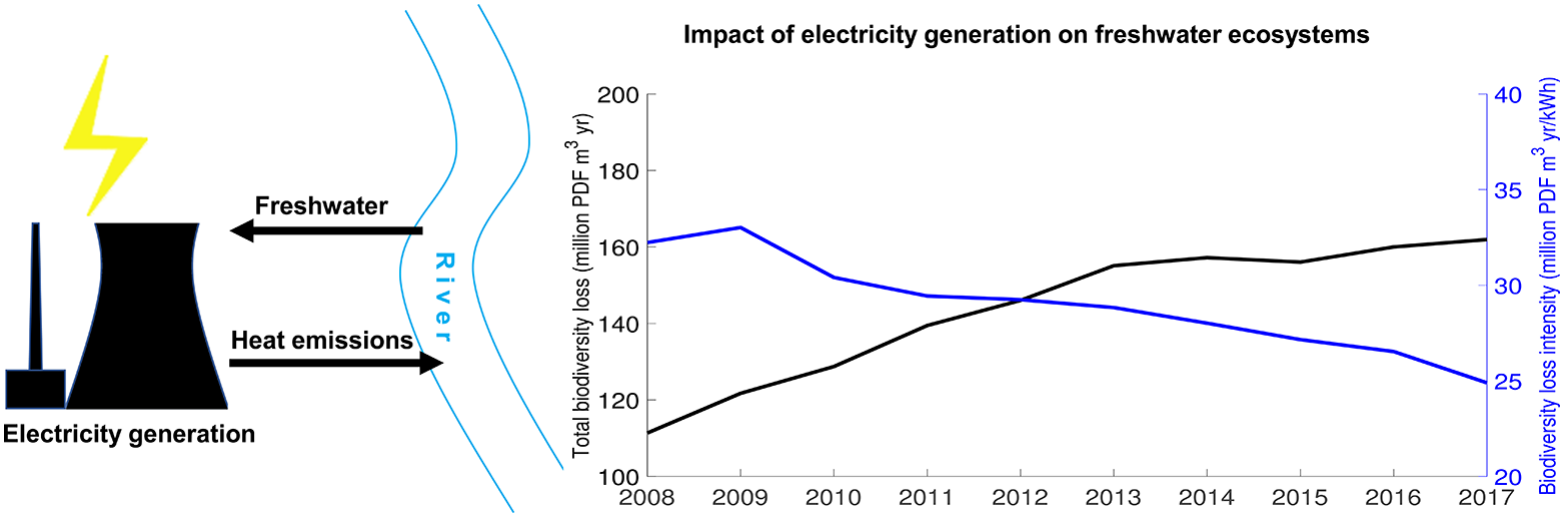

Electricity generation has two major, under-investigated impacts on freshwater biodiversity due to its water use: the consumption of freshwater and thermal emissions to freshwater. Here, we analyze the spatiotemporal freshwater biodiversity impacts of China’s electric power system and the driving factors for these impacts. We show that between 2008 and 2017, the freshwater consumption of electricity generation peaked in 2013 (13.6 Gm^3^). Meanwhile, the freshwater consumption factor of China’s electricity generation decreased from 3.2 to 2.0 L/kWh. However, due to increasing thermal emissions, the biodiversity loss via freshwater use increased from 1.1 × 10^8^ in 2008 to 1.6 × 10^8^ PDF m^3^ year. The overall biodiversity loss per unit of electricity generation decreased from 3.2 × 10^–5^ to 2.5 × 10^–5^ PDF m^3^ year/kWh. Biodiversity loss from thermal pollution is 60% higher than that driven by water consumption. Electricity transmission results in the shifting of biodiversity impacts across regions. The results show that 15% of total biodiversity loss was embedded in transmission networks. In terms of electrical power system drivers of biodiversity loss, the total generation was the main driving factor of the increase in loss (rather than shifts in generation type, for example). Our results indicate the necessity of assessing the biodiversity impacts of electricity generation and incorporating them into energy system planning.

## Abstract 2

Freshwater biodiversity impacts from freshwater consumption and thermal pollution for power production in China are increasing over time.

## 1 Introduction

While carbon emissions are a key environmental focus of electricity generation analyses, their biodiversity impacts have been largely overlooked.^1−5^ Biodiversity is a critical indicator of ecosystem health and provides many ecosystem services to society.^6^ Human activities are causing an accelerating biodiversity loss at rates 100–1000 times prehuman levels.^7^ Current losses in biodiversity are considered critical and could threaten earth system functioning and its adaptive capacity.^8^ Simultaneously, global electricity generation is growing quickly, dominated by thermal power (77% of the total) and hydropower (16%) in 2018.^9^ Linking electricity generation with biodiversity impacts can help deepen the understanding of biodiversity conservation and energy transition.

Current electrical power systems require large amounts of freshwater in the thermodynamic conversion of heat to work or the water held in hydropower reservoirs. These processes can result in both consumption of water or the warming of water in the environment (termed thermal emissions).^10−12^ Both freshwater consumption and thermal emissions have impacts on biodiversity^13,14^ (water consumption refers to the volume of water not returned to the water body due to evaporation, transpiration, or incorporation into products^10^). Research has shown that thermal and hydropower generators are major water consumers. Emerging renewables such as wind power and photovoltaic (PV) consume negligible water during operation.^15^

Different power-generating technologies use water in different ways. Thermal power plants withdraw water for cooling,^16,17^ and some of the water is consumed through evaporation.^18,19^ Liao et al.^20^ and Zhang et al.^21^ assessed the freshwater consumption of China’s thermal power production and found freshwater consumption of 3.8 and 5.7 Gm^3^ in 2010 and 2015, respectively. While hydropower is an important renewable energy source, it can consume a lot of water via evaporation from the reservoir surface.^22,23^ Estimates of water use for hydropower range widely.^24^ For China, Liu et al. showed the water intensity of hydropower plants ranging from 13 to 15 244 m^3^ MW h^–1^.^25^ Zhu et al.^26^ and Liao et al.^20^ found that 11.5–14.6 Gm^3^ of freshwater was consumed for China’s hydropower production in 2010.

Despite the large water requirements of electricity generation, the aquatic biodiversity impacts of electricity generation have received little attention. Dorber et al., in the few examples of such an assessment, quantified the water consumption of Norwegian hydropower reservoirs and found that the impacts on fish species vary over 6 orders of magnitude.^27^ Biodiversity impacts of electricity generation in China are of specific interest, as the two most biodiversity-threatening generation types, thermal and hydropower, together comprise 87% of national electricity generation (as of 2019^28^). However, their water consumption-related biodiversity impacts have not been quantified in previous research.

In addition to water consumption, the heat transferred into cooling water from power plants and then returned to the water source also has biodiversity impacts.^29,30^ For thermal power, there are two common wet cooling types: (1) once-through cooling, requiring large amounts of water withdrawal and directly returning most of that water to its source and (2) closed-loop cooling, in which some of the water is consumed through evaporation.^31^ Freshwater heat pollution is predominately from once-through cooling systems, which involves the direct rejection of the heat back into the water body.^12,32^ The temperature of discharged water from plants is higher than the natural river temperature and harmful to aquatic systems.^33,34^ In closed-loop cooling, almost all of the heat absorbed during the steam cycle is removed via evaporation and dissipated into the atmosphere. The heat contained in the periodic cooling tower blowdown is negligible compared to the heat released in once-through cooling emissions.^35^ Raptis et al. assessed the biodiversity loss caused by freshwater thermal pollution and showed the varying impact of electricity generation between countries.^12^ Pfister and Suh assessed the impact of thermal pollution on freshwater ecosystems in the U.S., finding that the ecosystem impact for the different U.S. electricity grids can differ by an order of magnitude.^36^ Cheng et al. simulated the impacts of thermal pollution from power plants on the aquatic ecosystem, indicating that fishes can be heavily affected.^37^ Hydropower stations also increase the temperature of the rejected water, but to a lesser degree than thermal power, so its impact on aquatic biodiversity was often neglected. The overall impact of thermal pollution from China’s power production on aquatic biodiversity has not been fully understood.

Here, we assess the impacts of both water consumption and thermal pollution for power production on freshwater biodiversity for the first time. We also extend the research to include hydropower. As with commodity trade, exchanges of electricity across large grids can result in the shifting of biodiversity impacts across regions. While international commodity trade can have significant biodiversity impacts (17–30% of global biodiversity loss^38^), China exchanges very little electricity internationally. However, the scale of interprovincial power transmission within China is large and increasing (with a 150% growth over 2008–2017). Electricity importers across China are outsourcing biodiversity impacts via transmission to other provinces, and we capture these dynamics. Finally, we diagnose the driving factors for changes in biodiversity loss over time (including generation type, the scale of electrical generation, and others).

## 2 Methods and Materials

### 2.1 Methods

**Figure 1:**
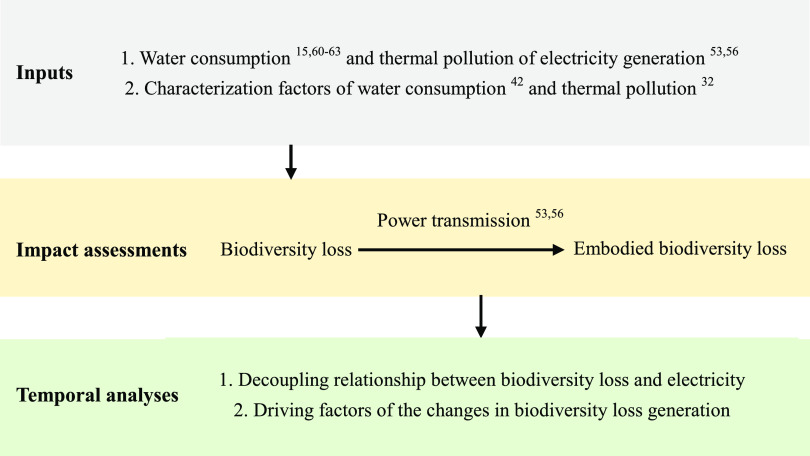
Overall schematic of the model.

The overall modeling approach is shown in Figure 1. First, we prepare the input data for assessments (gray box), i.e., the water consumption and thermal pollution of electricity generation and province-level, generator-specific characterization factors. We then assess the biodiversity loss caused by electricity generation along with the embodied biodiversity loss via power transmission (yellow box). Based on these calculations, we examine the relationships between biodiversity loss and electricity generation along with the driving factors of biodiversity loss via electricity generation (green box).

#### 2.1.1 Use of Water for Electricity Generation

We assess the provincial water consumption factors for thermal power and hydropower generation using the method described in Jin et al.^10^ Our database covers 96 and 50% of the national installed capacity for thermal power and hydropower, respectively. Thermal plant information included: plant name, installed capacity, the beginning year of operation, unit type, location, operation status, cooling system, and monthly electricity generation. Hydropower information included: plant name, installed capacity, year of operation start, location, operation status, reservoir area, and electricity generation. These representative plants are used to assess provincial water intensities (capacity-weighted water consumption of plants), which are then combined with provincial power production to assess water consumption. The total water consumption of electricity generation in each year is calculated as follows
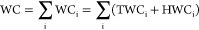
1where WC is the national water consumption for electricity generation (m^3^); WC_i_ is the water consumption for electricity generation in province i (m^3^); TWC_i_ is the water consumption for thermal power generation in province i (m^3^); and HWC_i_ is the water consumption for hydropower generation in province i (m^3^). For further details, see Supporting Information S1.

#### 2.1.2 Biodiversity Loss

Among the three main types of ecosystems (terrestrial, freshwater, and marine), we focus on biodiversity impacts in freshwater ecosystems, as much of the impact of water use inland is on freshwater systems.^39^ We consider water consumption and water thermal pollution of electricity generation as drivers of biodiversity loss. Freshwater consumption results in reduced river discharge, which is one of the main threats to freshwater life.^40^ The impacts can be assessed based on the species–discharge relationship. We consider fishes, given that this species group is larger than most other freshwater taxa,^41^^41^ and they are better studied. Water consumption is translated into impacts on freshwater biodiversity using characterization factors (CFs) expressed as a potentially disappeared fraction of species (full unit: PDF m^3^ year/m^3^) (Supporting Information S2).^42^ The increased river temperature caused by thermal pollution damages the ingestion and health of freshwater life and can lead to death.^33^ The impacts can be assessed based on species sensitivity distributions, considering the temperature tolerance interval of aquatic species (among which we include fishes, mollusks, crustaceans, and annelids).^12^ Thermal pollution is calculated and translated into impacts by CFs with the unit of PDF m^3^ year/MJ^12^ (Supporting Information S3). Ecosystem impacts refer to the fraction of species that is committed to becoming extinct (“potentially disappeared fraction of species” or PDF) if the pressure (e.g., water consumption) continues.^39^ As there are typically lag times between the pressure and the effect, the duration of the pressure influences whether the full extent of the effect will happen or not. For this reason, the exposure duration (year) to the pressure is also included in the unit of ecosystem impacts. Furthermore, impacts are related to the system being affected; here, the volume of water (m^3^). Hence, impact scores can be interpreted as an increase in extinction risk in a system over a certain exposure period. By multiplying these characterization factors (CFs) with the inventory flows (m^3^ in the case of water consumption and MJ for thermal pollution), we find the ecosystem impact scores for different impact categories measured in PDF m^3^ year.

The total freshwater biodiversity loss caused by electricity generation is calculated as follows
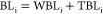
2where BL_i_ is the biodiversity loss caused by electricity generation in province i (PDF m^3^ year); WBL_i_ is the biodiversity loss caused by water consumption for electricity generation in province i (PDF m^3^ year); and TBL_i_ is the biodiversity loss caused by thermal pollution from electricity generation in province i (PDF m^3^ year).

Based on the results of provincial biodiversity loss via electricity generation, we examine the biodiversity loss embodied in power transmission, given by
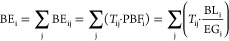
3where BE_i_ is the total biodiversity loss embodied in the power transmission from province i to other provinces (PDF m^3^ year); BE_ij_ is the biodiversity loss embodied in the power transmission from province i to j (PDF m^3^ year); *T*_ij_ is the power transmission from province i to j (GWh); PBF_i_ is the biodiversity loss per unit of electricity generation in province i (PDF m^3^ year/GWh); and EG_i_ is the total electricity generation in province i (GWh).

The net outsourcing of biodiversity loss can be obtained for each province with
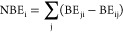
4where NBE_i_ is the net outsourcing of biodiversity loss of province i (PDF m^3^ year). If the NBE_i_ is positive, province i is a beneficiary of power transmission.

#### 2.1.3 Decoupling between Biodiversity Loss and Electricity Generation

Analyzing the decoupling of environmental impacts from their driving forces can help to identify the trends in impacts for policymakers.^43^ A widely used model proposed by Tapio^44^ decouples relationships between various environmental impacts and their drivers.^45−48^ Here, we use the Tapio model to examine the decoupling between biodiversity loss and electricity generation, with the decoupling degree (θ_*t*_) calculated by
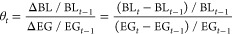
5where subscript *t* refers to the target year; ΔBL is the change in biodiversity loss during (*t* – 1, *t*); and ΔEG is the change in electricity generation during (*t* – 1, *t*). The decoupling state quadrant map corresponding to the decoupling indicator is shown in Figure S1.

#### 2.1.4 Decomposition Analysis of Biodiversity Loss

To assess the driving factors of environmental impacts, we apply Logarithmic Mean Divisia Index (LMDI) decomposition.^49^ LMDI has no residuals and is transparent in the interpretation of results.^47,50^ We decompose the driving factors as
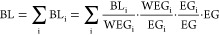
6where WEG_i_ is the water-using electricity generation (hydropower and thermal power) in province i (GWh); EG_i_ is the total electricity generation in province i (GWh); EG is the national electricity generation (GWh); BL_i_/WEG_i_ represents the biodiversity loss per unit of electricity generation using freshwater during its operation (hydropower and thermal power) in province i; WEG_i_/EG_i_ represents the proportion of water-using electricity generation in province i; EG_i_/EG represents the proportion of the electricity generation of province i in the national electricity generation; and EG represents the national electricity generation. Set
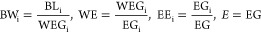
Equation 6 can be transformed into
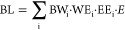
7where the potential driving factors are: (1) BW_i_, representing the biodiversity loss intensity of electricity generation; (2) WE_i_, representing the structure of electricity generation; (3) EE_i_, representing the distribution of electricity generation; and (4) *E*, representing the scale of electricity generation.

The two LMDI approaches, additive decomposition and multiplicative decomposition, can be related to one another using several expressions.^51^ In this study, the additive decomposition method is used to analyze the effects of biodiversity loss intensity, electricity generation structure, electricity generation distribution, and electricity generation scale on biodiversity loss during 2008–2017. The total biodiversity loss from the beginning period (base period) to *t*, the final period (report period) can be expressed as
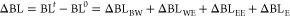
8Four effects of biodiversity loss changes are modeled: the biodiversity loss intensity effect (ΔBL_BW_), the electricity generation structure effect (ΔBL_WE_), the electricity generation distribution effect (ΔBL_EE_), and the effect of electricity generation scale (ΔBL_E_).

The decomposition equations for each effect are shown as follows
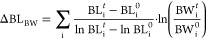
9
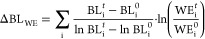
10
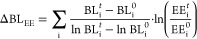
11
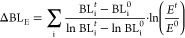
12

### 2.2 Materials

#### 2.2.1 Power Generation

This study includes 31 provincial-level administrative regions (provinces, autonomous regions, and municipalities; for simplicity, they are referred to as provinces, and their names are given in Figure S2). Provincial power generation during 2008–2017 was obtained from China Electric Power Yearbook^52^ and China Electricity Council.^53^ We focus on hydropower and coal-fired thermal power in this study as the major users of freshwater. Nuclear power is not included as plants in China are along the coastline and use seawater for cooling, which would impact marine environments rather than the freshwater environments we assess here.^15,54^ Coal, hydropower, and nuclear power dominate power production, while gas and oil power plants account for less than 5% of the total during the study period, and they are not included due to data limitations.^52^ The operational water consumption of wind and photovoltaic power is negligible and thus not considered. Other electricity-generating technologies accounted for less than 7% of the total during the study period and did not discharge freshwater thermal pollution to rivers.^32,55^

#### 2.2.2 Power Transmission

Interprovincial power transmission during 2008–2017 was obtained from the China Electricity Council.^56^ These data are mostly reported in the form of province-to-province transmission. A small amount of transmission data is from provinces to the subnational grid. We disaggregate them into the province-to-province transmissions based on existing electricity transmission lines.^57,58^

#### 2.2.3 Water

Water consumption factors are obtained from Jin et al.,^15^ which assessed the provincial factors based on plant-level data in 2017. The national factors in 2008–2016 were reported by China Electricity Council.^59^ We assessed the provincial factors by assuming that they changed in proportion to the national factors. The water consumption factors for hydropower are not reported in this data set, so the data from Jin et al.^15^ were used. The provincial water availability and water use were obtained from the Ministry of Water Resources^55,60−63^ and used to assess water stress and Characterization factors (Supporting Information S5).

## 3 Results

### 3.1 Electric Power System and Its Water Use

**Figure 2:**
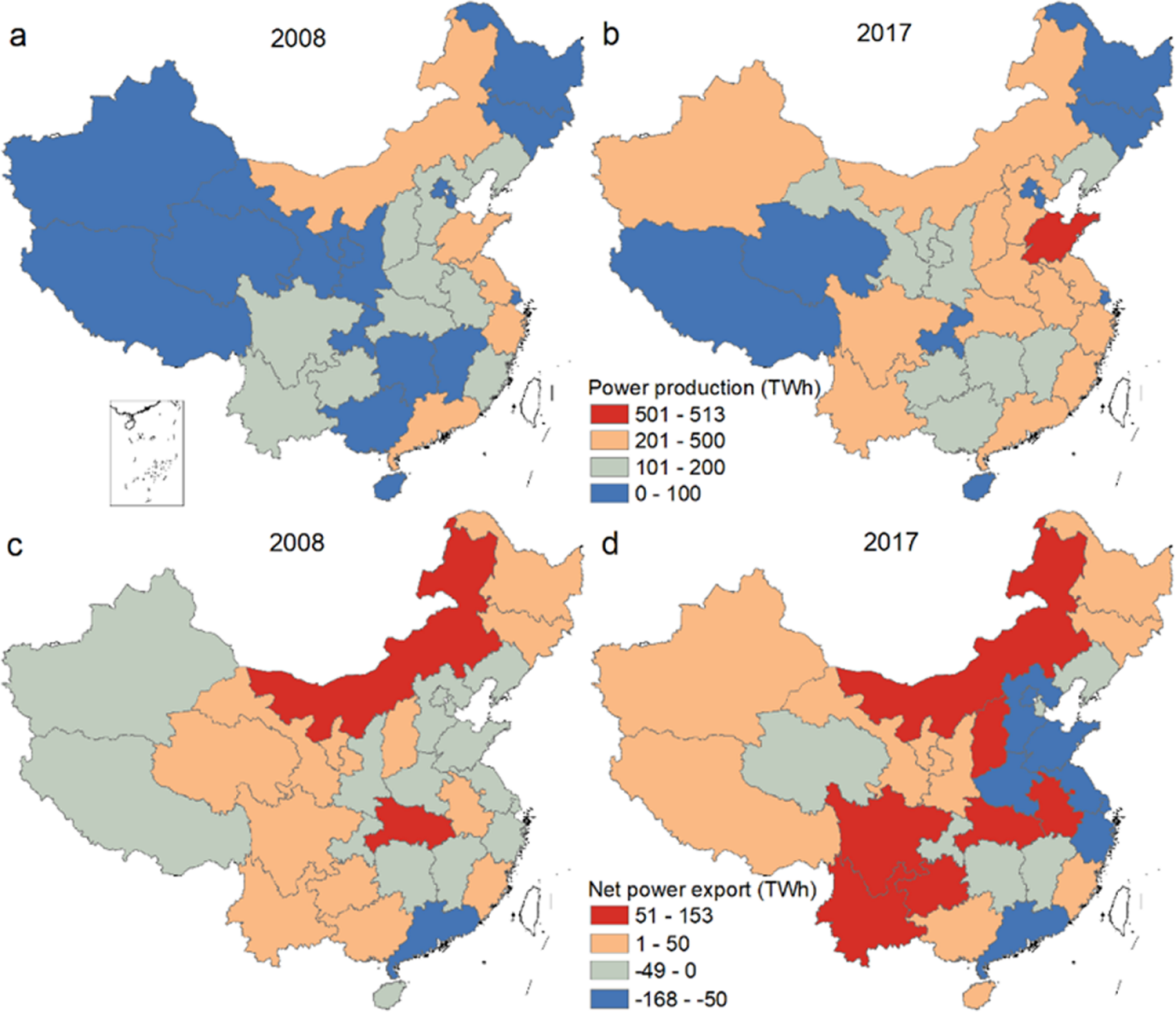
Provincial power production (a, b) and net power exports (c, d) in 2008 and 2017.

Nationally, electricity generation almost doubled between 2008 and 2017, from 3451 to 6417 TWh (Figure S3). The increase was slowest in 2015 (2.4%) and fastest in 2017 (6.5%). Coal power grew continuously and was the largest contributor to the total generation increase throughout the period, but its share in the total electricity generation decreased to 65% by 2017. Wind and solar power developed quickly but still accounted for only 5 and 2% of the total, respectively, by 2017. All provinces saw an increase in power production during 2008–2017, while many coastal regions experienced an increase in power imports (Figure 2). Shandong province is the largest electricity producer (513 TWh in 2017, 95% of which was from thermal power). Sichuan province is the top hydropower producer (304 TWh in 2017).

**Figure 3:**
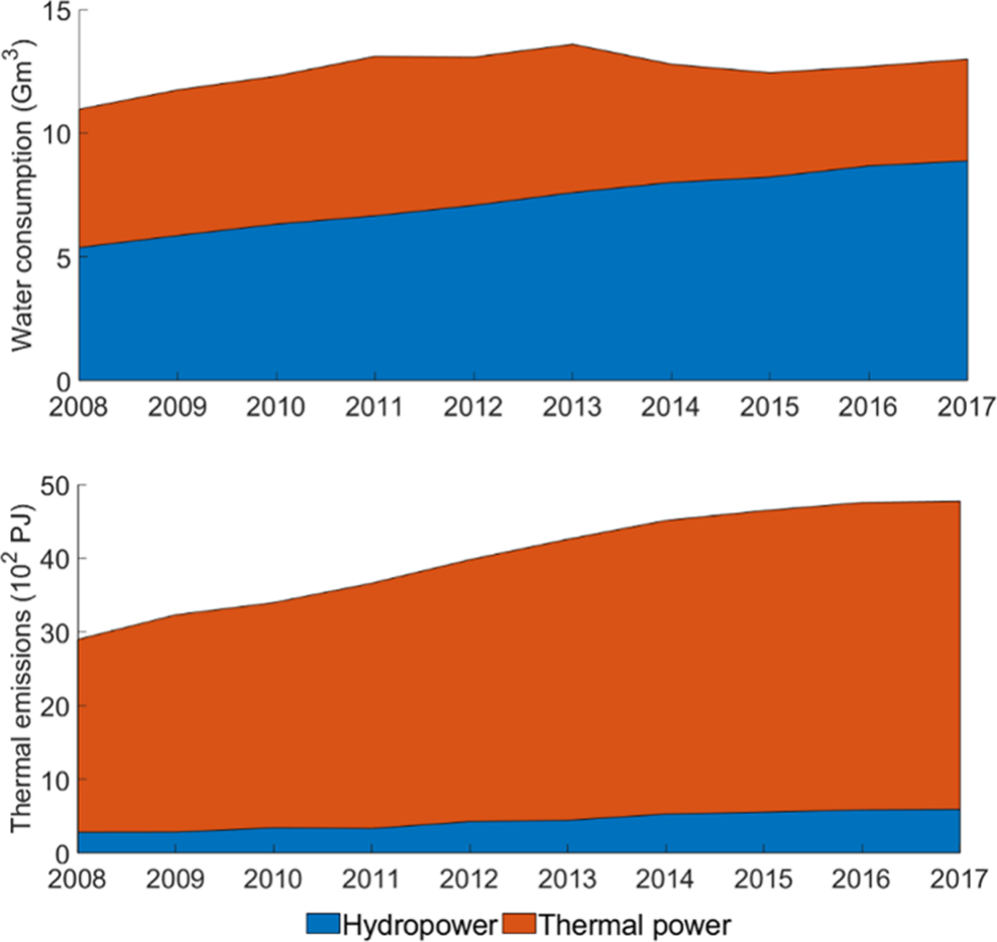
Water consumption and thermal emissions of electricity generation in China during 2008–2017.

During 2008–2017, national freshwater consumption of electricity generation peaked in 2013 (13.6 Gm^3^) and declined to 12.4 Gm^3^ in 2015 (Figure 3). However, freshwater consumption began rising again in 2016 due to hydropower expansions and stagnation in previous improvements in thermal water intensities. In 2017, total freshwater consumption for electricity generation was 13 Gm^3^. Thermal power water consumption peaked in 2011 (6.5 Gm^3^) and then declined to 4.1 Gm^3^ in 2017. Water consumption of hydropower increased continuously and reached 8.9 Gm^3^ in 2017. Electricity generation accounted for 34% of the total industrial freshwater consumption in 2008, with the proportion rising to 43% in 2017. Hunan province was the largest consumer, with a freshwater consumption of 1.2 Gm^3^, whereas Beijing consumed the least (0.02 Gm^3^). The freshwater consumption factor of China’s electricity generation decreased from 3.2 to 2.0 L/kWh during 2008–2017. Tibet generated electricity with the highest water consumption factor (9.7 L/kWh in 2017), as it relies on hydropower. Shanghai, with once-through cooling systems for thermal power and no hydropower, had the lowest water consumption factor of 0.46 L/kWh in 2017.

The average annual freshwater thermal emission of power production was 2996 and 4771 PJ in 2008 and 2017, respectively. Thermal power accounted for ∼90% of the total thermal emissions, while the remaining 10% is from hydropower due to its cooling needs. Jiangsu province is the largest emitter of thermal pollution due to its use of once-through cooling systems.

### 3.2 Biodiversity Impacts of Electricity Generation

**Figure 4:**
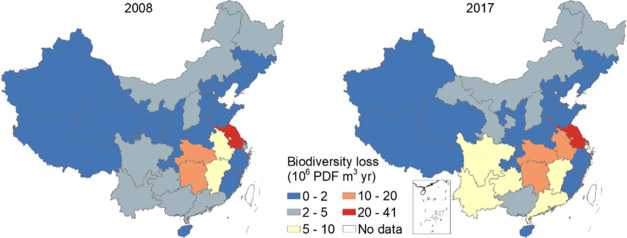
Provincial freshwater biodiversity loss caused by electricity generation in 2008 and 2017.

The total biodiversity loss by water consumption and thermal pollution of China’s electricity generation increased from 1.1 × 10^8^ in 2008 to 1.6 × 10^8^ PDF m^3^ year in 2017 (Figure S4). Thermal power accounted for 72 and 65% of the total biodiversity loss of power production in 2008 and 2017, respectively. The impact of thermal power peaked in 2013, whereas the impact of hydropower kept increasing during the study period. Despite the increase of the total impact, the biodiversity loss per unit of electricity generation reduced from 3.2 × 10^–5^ to 2.5 × 10^–5^ PDF m^3^ year/kWh. Compared to thermal power (2.3 × 10^–5^ PDF m^3^ year/kWh), hydropower (4.7 × 10^–5^ PDF m^3^ year/kWh) caused double the biodiversity loss per unit of electricity produced in 2017 because of its higher water consumption. The impact of freshwater thermal emission (1 × 10^8^ PDF m^3^ year in 2017) is 60% larger than that of freshwater consumption (6.2 × 10^7^ PDF m^3^ year in 2017). In China, the south generally faced larger biodiversity impacts than the north (Figure 4). Jiangsu, Hunan, Hubei, and Anhui provinces alone contributed to 57% of the biodiversity loss of power production in 2017.

### 3.3 Biodiversity Impacts Embodied in Power Transmission

The interprovincial electricity transmission increased more rapidly than electricity generation, from 445 TWh in 2008 to 1130 TWh in 2017 (Figure S5). The transmission is mainly from the west to the east. Inner Mongolia is the largest electricity exporter (exporting 55 TWh), whereas Guangdong was the largest electricity importer (importing 185 TWh) in 2017. During 2008–2017, embodied thermal pollution via power transmission increased from 12.6 to 17.9 GW, and embodied water increased from 1.5 to 2.0 Gm^3^. Across the country, 17 provinces were net water exporters, while 14 provinces were net importers in 2017. There were 15 water-scarce provinces (water stress index larger than 0.5), of which 47% were net water exporters with a contribution of 23% to the total electricity generation.

**Figure 5:**
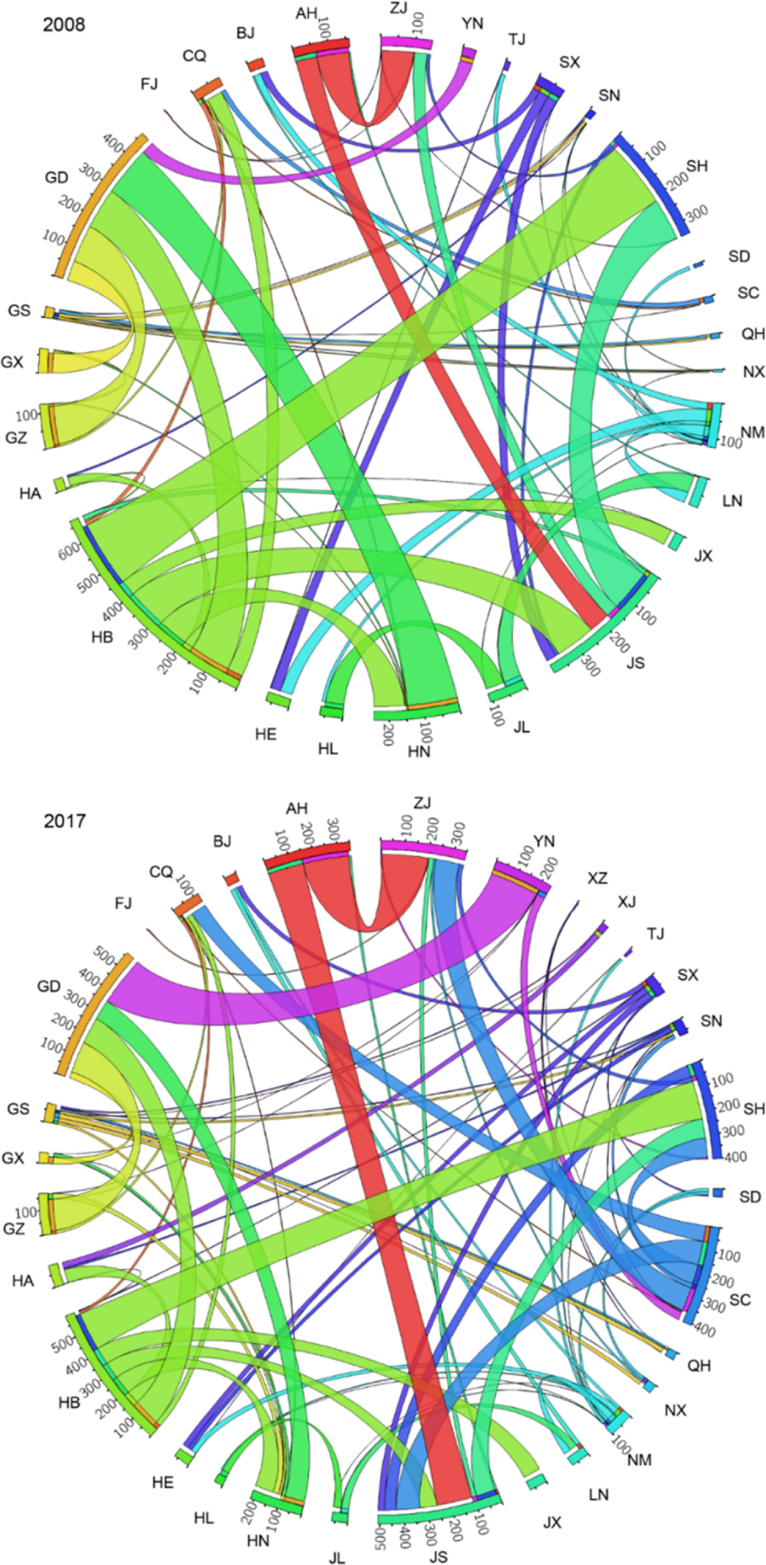
Biodiversity loss embodied in interprovincial power transmission in 2008 and 2017. Each color represents an exporting region. Numbers are in the unit of 10^4^ PDF m^3^ year. Please see the provinces’ full names and abbreviations in Table S2.

Power transmission accounted for 15% of total biodiversity loss of power production in 2017. The biodiversity loss embodied in interprovincial power transmission increased by 39% during 2008–2017. Guangdong (GD) province was the largest beneficiary in both 2008 and 2017 by importing a large amount of electricity, with a net import of biodiversity of 4.5 × 10^6^ and 5.4 × 10^6^ PDF m^3^ year, respectively. Hubei (HB) province was the largest net exporter of biodiversity in both 2008 and 2017 (Figure 5).

### 3.4 Trends and Driving Factors of Biodiversity Impacts

We see an overall decoupling between biodiversity loss and electricity generation during the study period (Table S3). There was a 45% increase in biodiversity loss and an 88% increase in power production during 2008–2017. During 2011–2013, biodiversity loss and electricity generation experienced an expansive coupling because of the increase in thermal pollution from thermal power. However, their relationship turned back into decoupling after 2013 due to the slow increase or even decrease in biodiversity impacts.

**Figure 6:**
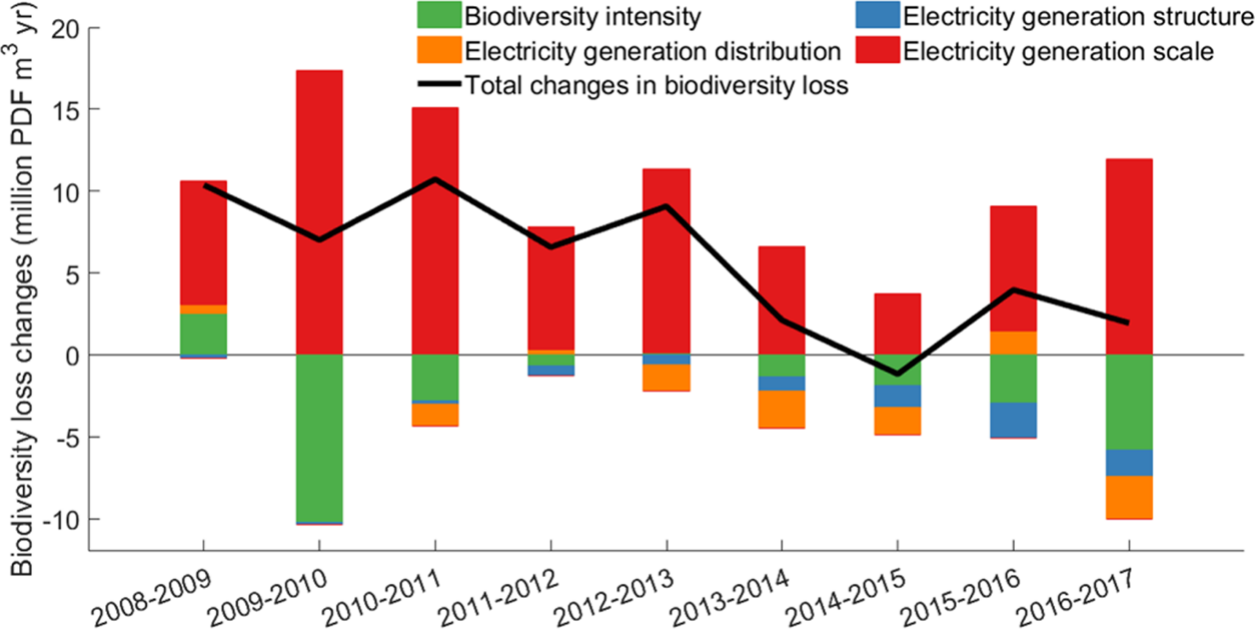
Decomposition of the changes in biodiversity loss during 2008–2017.

During the study period, the increases in biodiversity loss each year from electricity generation slowed (see Figure 6). The expansion of electricity generation (the scale parameter in the driving forces) was the main driving factor of the increase of biodiversity loss, whereas the biodiversity loss intensity saw decreases and lowered overall biodiversity loss (Figure 6). The impact of the electricity generation scale generally decreased from 2011 to 2015 but began to rise in 2016. The electricity generation structure change, i.e., the decrease of the share of freshwater-using electric power (hydropower and thermal power) in total generation, had a positive but relatively small effect on biodiversity conservation. Although the amount of freshwater-using electric power did not see a decrease, this effect still has increased in recent years due to the increases in wind, solar, and nuclear power. In fact, hydropower and thermal power have seen a continual increase since 2011. From the perspective of the cumulative impact, 22 provinces saw an increase in biodiversity loss, whereas 9 saw a decrease. Jiangsu province was the largest contributor to biodiversity loss due to increases in electricity generation, whereas Heilongjiang province was the largest contributor to reducing biodiversity loss due to the decrease in biodiversity loss intensity of electricity generation and proportion in the national electricity generation.

## 4 Discussion

### 4.1 Energy Transition and Biodiversity Impacts

China’s total electricity generation grew continuously over the study period, with a remarkable change in the electricity generation structure toward wind and solar power. Meanwhile, hydropower and thermal power generation also increased by 111 and 63% during 2008–2017, respectively, keeping water consumption high throughout the period. Hydropower is expected to increase,^64^ indicating the strong possibility of an increase in hydropower-related biodiversity impacts in the future. Recently, China has proposed strict regulations on the water use of thermal power, but these have not been formally adopted yet.^65,66^ There has been a program of shutting down small and inefficient thermal power plants while constructing supercritical and ultra-supercritical units, all of which have saved water.^67^ Additionally, there are two classes of air cooling: direct air cooling and indirect air cooling.^31^ The indirect air-cooling systems, where the condenser system uses water in its cycle but without any evaporation, are increasingly used in water-scarce regions in China.^15^ These systems have the advantage of both direct air cooling (low water intensity) and wet cooling (stable cooling efficiency).^68^ Many of the easiest implemented water-saving technologies have already been widely adopted, and the potential for further improvements is diminishing (with a reduction in water consumption factor of only 0.02 L/kWh per year during 2017–2019).^21,59^

Decomposition results show that the structure and distribution of electricity generation had a small overall reducing effect on biodiversity loss, indicating that electricity generation has shifted toward low biodiversity-impact regions and technologies. Electricity transmission has promoted the development of wind, solar, and hydropower in western and northern China. Its continued expansion, along with market developments, will enable further optimization of power structure and distribution. However, its impact on biodiversity loss is uncertain and depends on the choices made between water-using and other energy technologies.

### 4.2 Comparison with Previous Studies

Pfister and Suh assessed the impact of thermal emissions from electric power generation on freshwater ecosystems in the U.S., finding that less than 5% of values are below 1.0 × 10^–5^ PDF m^3^ year/kWh and less than 0.1% above 1.0 × 10^–3^ PDF m^3^ year/kWh.^36^ Raptis et al. showed that the thermal emissions impact of China’s electricity generation in 2011 was 4.0 × 10^7^ PDF m^3^ year.^12^ Our results showed that the impact was 6.9 × 10^7^ PDF m^3^ year in 2011 and then increased to 8.6 × 10^7^ PDF m^3^ year in 2017. The differences between Raptis et al. and our results arise mainly from two sources: the lower coverage of thermal power and the lower capacity factors in Raptis et al.^,^^12^ which are based on data from the U.S. Energy Information Administration. In addition to thermal pollution, water consumption is another major cause of biodiversity impact. We extended previous thermal power studies to include the water consumption of both hydropower and thermal power. Results show that the impact of freshwater consumption was smaller than thermal emissions. Previous studies have not quantitatively analyzed the temporal changes and driving factors of biodiversity impacts. Our analysis indicated an overall relative decoupling between electricity generation and biodiversity impacts. The expansion of the electricity generation scale and the decrease in biodiversity loss intensity of electricity generation were identified as the major driver and preventer of biodiversity loss, respectively.

This study focused on China; in the future, it will be important to make assessments for other nations or on a global scale. While local, regional, and global species losses are relevant, only global losses cannot be recovered. Unfortunately, local or regional relative species loss cannot be easily aggregated or compared on a global level or against other estimates for several reasons. First, the same relative species loss can imply very different absolute species losses in different regions. Second, some regions host more endemic species than others. It is more likely that regional losses in those regions lead to global extinctions than in regions associated with fewer endemic species. We used conversion factors to convert regional species richness impacts into potential global species extinctions.^69^ Our results showed that the global impacts increased from 1.0 × 10^–4^ to 1.6 × 10^–4^ PDF year during 2008–2017 (Supporting Information S6). The biodiversity impacts were expressed as the potentially disappeared fraction of species (PDF) caused by water use (freshwater consumption and thermal emissions). In the future, assessments should be conducted for a broader range of impact and sector categories than just water use of electricity generation as done in this study, which will allow a better understanding of anthropogenic impacts on biodiversity.

### 4.3 Limitations and Implications

In this study, we focused on operational water use rather than life cycle water use. The fuel cycle and plant infrastructure may require large amounts of freshwater, depending on the fuel type.^10^ Further work could focus on the biodiversity impacts of life cycle water use in the future when data are available, i.e., the location and way of fuel mining activities, the materials of plant infrastructure, and their sources. For thermal power plants, the use of carbon capture and storage (CCS) in the future to meet climate targets will pose a threat to water-related biodiversity, as it heavily relies on water resources.^70,71^ However, the potential impact of CCS was not considered in this study because of the lack of information on the location and scale of CCS deployment in the future. In addition, this study focused on the water-related biodiversity impacts, but the biodiversity loss of other pressures from electricity generation are not included. For example, the land occupation by solar power and windfarms^72,73^ and the freshwater habitat fragmentation^74^ and flow alterations^24^ caused by hydropower dams have impacts on biodiversity. An impact assessment of habitat fragmentation would require the development of new characterization factors. The species–discharge relationship used to assess impacts from freshwater consumption does not consider impacts from flow alterations, of which increased discharges also can have adverse impacts on freshwater biodiversity. Such flow alterations have so far only been considered within water stress footprints,^24^ but no characterization factors exist yet that extend the cause–effect chain to biodiversity impacts. Freshwater biodiversity is complex, and the species richness pattern of one taxon is unlikely to be a good indicator of the pattern of another taxon.^75^ While we considered four species groups for the impacts of thermal pollution, we focused only on fishes for the impacts of freshwater consumption. Future studies could expand the taxonomic coverage for freshwater consumption impact assessment when related data and models become available. Additionally, the species–discharge relationship would benefit from regionalization^76^ to account for factors such as different climatic conditions. The thermal pollution impacts on river temperature and biodiversity may differ across different types of outfalls of power plants.^37^ It will be of interest to distinguish outfall types when data become available. There are ∼47 000 hydropower plants in China,^77^ of which we only cover about half. This results in uncertainties, as water use differs a lot across hydropower plants.

According to these results, we make several suggestions for mitigating the impacts of the electric power system on freshwater biodiversity. First, it is important to reduce the water use of hydropower and thermal power, as they dominate the current energy system. Our results showed that the water consumption of hydropower has large impacts on biodiversity and is expected to increase in the future, indicating the necessity to build run-of-river hydropower plants (a type of hydroelectric generation plant that has little or no water storage and reservoir evaporation). For thermal power, adopting air cooling systems and using seawater and reclaimed water for cooling are feasible and effective ways of reducing freshwater demand. Air-cooling systems are commonly used by newly built plants. Indeed, 29% of operating plants now use this technology, indicating the potential for further reducing water requirements if this proportion was to increase. Seawater use in coastal regions (such as Jiangsu, Shanghai, and Guangdong) is encouraged by the government.^15^ We show that 15 billion m^3^ of freshwater can be saved by switching to seawater cooling for power plants near the coast (within 10 km). However, the economic costs of retrofitting cooling systems and building seawater treatment facilities need more research. Since hydropower is a renewable resource that can enable greater amounts of other renewables in the electricity system (via the provision of grid stability functions and load matching renewable variations), the net result of associated climate-change-driven biodiversity loss through lower hydropower capacity and the freshwater biodiversity loss of hydropower water use is not straightforward. Second, the further development of renewables such as photovoltaics and wind power is crucial since both consume a negligible amount of water. Under the International Energy Agency’s (IEA’s) sustainable development scenario, China’s wind and solar PV will experience a rapid increase by 2439 TWh through the period 2017–2030, equal to 42% of the total hydro and thermal power production in 2017. This suggests a significant opportunity in switching to a low water intensity power system.^64^ Third, we can shift electricity generation from regions with high biodiversity intensities to those with low biodiversity intensities by considering the provincial biodiversity factors of electricity generation assessed in this study.

## Supporting Information Available

More detailed documentation of methods and additional results (PDF)

## References

[ref1] Lèbre l.; Stringer M.; Svobodova K.; Owen J. R.; Kemp D.; Côte C.; Arratia-Solar A.; Valenta R. K. The social and environmental complexities of extracting energy transition metals. Nat. Commun. 2020, 11, 4823 10.1038/s41467-020-18661-9. 32973153 PMC7519138

[ref2] Peng W.; Wagner F.; Ramana M. V.; Zhai H.; Small M. J.; Dalin C.; Zhang X.; Mauzerall D. L. Managing China’s coal power plants to address multiple environmental objectives. Nat. Sustainabiity 2018, 1, 693–701. 10.1038/s41893-018-0174-1.

[ref3] Popescu V. D.; Munshaw R. G.; Shackelford N.; Montesino Pouzols F.; Dubman E.; Gibeau P.; Horne M.; Moilanen A.; Palen W. J. Quantifying biodiversity trade-offs in the face of widespread renewable and unconventional energy development. Sci. Rep. 2020, 10, 7603 10.1038/s41598-020-64501-7. 32371910 PMC7200705

[ref4] Wang J.; Zhong H.; Yang Z.; Wang M.; Kammen D. M.; Liu Z.; Ma Z.; Xia Q.; Kang C. Exploring the trade-offs between electric heating policy and carbon mitigation in China. Nat. Commun. 2020, 11, 6054 10.1038/s41467-020-19854-y. 33247140 PMC7695859

[ref5] Mason N.; Ward M.; Watson J. E. M.; Venter O.; Runting R. K. Global opportunities and challenges for transboundary conservation. Nat. Ecol. Evol. 2020, 4, 694–701. 10.1038/s41559-020-1160-3. 32203481

[ref6] Harrison P. A.; Berry P. M.; Simpson G.; Haslett J. R.; Blicharska M.; Bucur M.; Dunford R.; Egoh B.; Garcia-Llorente M.; Geamănă N.; Geertsema W.; Lommelen E.; Meiresonne L.; Turkelboom F. Linkages between biodiversity attributes and ecosystem services: A systematic review. Ecosyst. Serv. 2014, 9, 191–203. 10.1016/j.ecoser.2014.05.006.

[ref7] Lenzen M.; Moran D.; Kanemoto K.; Foran B.; Lobefaro L.; Geschke A. International trade drives biodiversity threats in developing nations. Nature 2012, 486, 109 10.1038/nature11145. 22678290

[ref8] Steffen W.; Richardson K.; Rockström J.; Cornell S. E.; Fetzer I.; Bennett E. M.; Biggs R.; Carpenter S. R.; de Vries W.; de Wit C. A.; Folke C.; Gerten D.; Heinke J.; Mace G. M.; Persson L. M.; Ramanathan V.; Reyers B.; Sörlin S. Planetary boundaries: Guiding human development on a changing planet. Science 2015, 347, 1259855 10.1126/science.1259855. 25592418

[ref9] International Energy Agency. Key World Energy Statistics, 2021.

[ref10] Jin Y.; Behrens P.; Tukker A.; Scherer L. Water use of electricity technologies: A global meta-analysis. Renewable Sustainable Energy Rev. 2019, 115, 109391 10.1016/j.rser.2019.109391.

[ref11] Holland R. A.; Scott K. A.; Flörke M.; Brown G.; Ewers R. M.; Farmer E.; Kapos V.; Muggeridge A.; Scharlemann J. P. W.; Taylor G.; Barrett J.; Eigenbrod F. Global impacts of energy demand on the freshwater resources of nations. Proc. Natl. Acad. Sci. U.S.A. 2015, 112, E6707 10.1073/pnas.1507701112. 26627262 PMC4672781

[ref12] Raptis C. E.; Boucher J. M.; Pfister S. Assessing the environmental impacts of freshwater thermal pollution from global power generation in LCA. Sci. Total Environ. 2017, 580, 1014–1026. 10.1016/j.scitotenv.2016.12.056. 28024751

[ref13] Vörösmarty C. J.; Rodríguez Osuna V.; Cak A. D.; Bhaduri A.; Bunn S. E.; Corsi F.; Gastelumendi J.; Green P.; Harrison I.; Lawford R.; Marcotullio P. J.; McClain M.; McDonald R.; McIntyre P.; Palmer M.; Robarts R. D.; Szöllösi-Nagy A.; Tessler Z.; Uhlenbrook S. Ecosystem-based water security and the Sustainable Development Goals (SDGs). Ecohydrol. Hydrobiol. 2018, 18, 317–333. 10.1016/j.ecohyd.2018.07.004.

[ref14] Miara A.; Tarr C.; Spellman R.; Vörösmarty C. J.; Macknick J. E. The power of efficiency: Optimizing environmental and social benefits through demand-side-management. Energy 2014, 76, 502–512. 10.1016/j.energy.2014.08.047.

[ref15] Jin Y.; Behrens P.; Tukker A.; Scherer L. The energy-water nexus of China’s interprovincial and seasonal electric power transmission. Appl. Energy 2021, 286, 116493 10.1016/j.apenergy.2021.116493.

[ref16] Van Vliet M. T.; Wiberg D.; Leduc S.; Riahi K. Power-generation system vulnerability and adaptation to changes in climate and water resources. Nat. Clim. Change 2016, 6, 375 10.1038/nclimate2903.

[ref17] Lohrmann A.; Farfan J.; Caldera U.; Lohrmann C.; Breyer C. Global scenarios for significant water use reduction in thermal power plants based on cooling water demand estimation using satellite imagery. Nat. Energy 2019, 4, 1040–1048. 10.1038/s41560-019-0501-4.

[ref18] Nouri N.; Balali F.; Nasiri A.; Seifoddini H.; Otieno W. Water withdrawal and consumption reduction for electrical energy generation systems. Appl. Energy 2019, 248, 196–206. 10.1016/j.apenergy.2019.04.023.

[ref19] Gao X.; Zhao Y.; Lu S.; Chen Q.; An T.; Han X.; Zhuo L. Impact of coal power production on sustainable water resources management in the coal-fired power energy bases of Northern China. Appl. Energy 2019, 250, 821–833. 10.1016/j.apenergy.2019.05.046.

[ref20] Liao X.; Zhao X.; Hall J. W.; Guan D. Categorising virtual water transfers through China’s electric power sector. Appl. Energy 2018, 226, 252–260. 10.1016/j.apenergy.2018.05.132.

[ref21] Zhang C.; Zhong L.; Wang J. Decoupling between water use and thermoelectric power generation growth in China. Nat. Energy 2018, 3, 792–799. 10.1038/s41560-018-0236-7.

[ref22] Bakken T. H.; Killingtveit A.; Engeland K.; Alfredsen K.; Harby A. Water consumption from hydropower plants - review of published estimates and an assessment of the concept. Hydrol. Earth Syst. Sci. 2013, 17, 3983–4000. 10.5194/hess-17-3983-2013.

[ref23] Grubert E. A. Water consumption from hydroelectricity in the United States. Adv. Water Resour. 2016, 96, 88–94. 10.1016/j.advwatres.2016.07.004.

[ref24] Scherer L.; Pfister S. Global water footprint assessment of hydropower. Renewable Energy 2016, 99, 711–720. 10.1016/j.renene.2016.07.021.

[ref25] Liu J.; Zhao D.; Gerbens-Leenes P. W.; Guan D. China’s rising hydropower demand challenges water sector. Sci. Rep. 2015, 5, 11446 10.1038/srep11446. 26158871 PMC4648423

[ref26] Zhu X.; Guo R.; Chen B.; Zhang J.; Hayat T.; Alsaedi A. Embodiment of virtual water of power generation in the electric power system in China. Appl. Energy 2015, 151, 345–354. 10.1016/j.apenergy.2015.04.082.

[ref27] Dorber M.; Mattson K. R.; Sandlund O. T.; May R.; Verones F. Quantifying net water consumption of Norwegian hydropower reservoirs and related aquatic biodiversity impacts in Life Cycle Assessment. Environ. Impact Assess. Rev. 2019, 76, 36–46. 10.1016/j.eiar.2018.12.002.

[ref28] National Bureau of Statistics of China. Electricity Balance Sheet, 2019.

[ref29] Madden N.; Lewis A.; Davis M. Thermal effluent from the power sector: an analysis of once-through cooling system impacts on surface water temperature. Environ. Res. Lett. 2013, 8, 035006 10.1088/1748-9326/8/3/035006.

[ref30] Logan L. H.; Stillwell A. S. Probabilistic assessment of aquatic species risk from thermoelectric power plant effluent: Incorporating biology into the energy-water nexus. Appl. Energy 2018, 210, 434–450. 10.1016/j.apenergy.2017.09.027.

[ref31] Zhang X.; Liu J.; Tang Y.; Zhao X.; Yang H.; Gerbens-Leenes P. W.; van Vliet M. T. H.; Yan J. China’s coal-fired power plants impose pressure on water resources. J. Cleaner Prod. 2017, 161, 1171–1179. 10.1016/j.jclepro.2017.04.040.

[ref32] Raptis C. E.; Pfister S. Global freshwater thermal emissions from steam-electric power plants with once-through cooling systems. Energy 2016, 97, 46–57. 10.1016/j.energy.2015.12.107.

[ref33] Yang L. P.; Wang Y. J.; Liang H.; Wang Y. H.; Cui Y. X.; Chen Y. F. Influence of thermal drainage from power plants on environment. Sci. Technol. Innovation Herald 2018, 124–127.

[ref34] Xin-yu W.; Yi-chuan W.; Kun Z.; Yu-qin D.; Xiao-wei X.; Zhao-rong S. Review of Impact Assessments of Thermal Discharges from Power Plants on Aquatic Biota. J. Hydroecol. 2018, 39, 1–10.

[ref35] Raptis C. E.; van Vliet M. T. H.; Pfister S. Global thermal pollution of rivers from thermoelectric power plants. Environ. Res. Lett. 2016, 11, 104011 10.1088/1748-9326/11/10/104011.

[ref36] Pfister S.; Suh S. Environmental impacts of thermal emissions to freshwater: Spatially explicit fate and effect modeling for life cycle assessment and water footprinting. Int. J. Life Cycle Assess. 2015, 20, 927 10.1007/s11367-015-0893-8.

[ref37] Cheng Y.; Jiang Y.; Qu J.; Xue Z.; Wu K. Heat effect of warm water discharge from power plant on fish: with different discharge arrangements. Therm. Power Gener. 2017, 46, 101–106.

[ref38] Wiedmann T.; Lenzen M. Environmental and social footprints of international trade. Nat. Geosci. 2018, 11, 314–321. 10.1038/s41561-018-0113-9.

[ref39] Verones F.; Hellweg S.; Antón A.; Azevedo L. B.; Chaudhary A.; Cosme N.; Cucurachi S.; Baan L.; Dong Y.; Fantke P.; Golsteijn L.; Hauschild M.; Heijungs R.; Jolliet O.; Juraske R.; Larsen H.; Laurent A.; Mutel C. L.; Margni M.; Núñez M.; Owsianiak M.; Pfister S.; Ponsioen T.; Preiss P.; Rosenbaum R. K.; Roy P. O.; Sala S.; Steinmann Z.; Zelm R.; Van Dingenen R.; Vieira M.; Huijbregts M. A. J. LC-IMPACT: A regionalized life cycle damage assessment method. J. Ind. Ecol. 2020, 24, 1201–1219. 10.1111/jiec.13018.

[ref40] Xenopoulos M. A.; Lodge D. M.; Alcamo J.; Märker M.; Schulze K.; Van Vuuren D. P. Scenarios of freshwater fish extinctions from climate change and water withdrawal. Global Change Biol. 2005, 11, 1557–1564. 10.1111/j.1365-2486.2005.001008.x.

[ref41] Grosberg R. K.; Vermeij G. J.; Wainwright P. C. Biodiversity in water and on land. Curr Biol. 2012, 22, R900–R903. 10.1016/j.cub.2012.09.050. 23137680

[ref42] Hanafiah M. M.; Xenopoulos M. A.; Pfister S.; Leuven R. S. E. W.; Huijbregts M. A. J. Characterization Factors for Water Consumption and Greenhouse Gas Emissions Based on Freshwater Fish Species Extinction. Environ. Sci. Technol. 2011, 45, 5272–5278. 10.1021/es1039634. 21574555

[ref43] Naqvi A.; Zwickl K. Fifty shades of green: Revisiting decoupling by economic sectors and air pollutants. Ecol. Econ. 2017, 133, 111–126. 10.1016/j.ecolecon.2016.09.017.

[ref44] Tapio P. Towards a theory of decoupling: degrees of decoupling in the EU and the case of road traffic in Finland between 1970 and 2001. Transp. Policy 2005, 12, 137–151. 10.1016/j.tranpol.2005.01.001.

[ref45] Li L.; Shan Y.; Lei Y.; Wu S.; Yu X.; Lin X.; Chen Y. Decoupling of economic growth and emissions in China’s cities: A case study of the Central Plains urban agglomeration. Appl. Energy 2019, 244, 36–45. 10.1016/j.apenergy.2019.03.192.

[ref46] Xie P.; Yang F.; Mu Z.; Gao S. Influencing factors of the decoupling relationship between CO2 emission and economic development in China’s power industry. Energy 2020, 209, 118341 10.1016/j.energy.2020.118341.

[ref47] Chen J.; Wang P.; Cui L.; Huang S.; Song M. Decomposition and decoupling analysis of CO2 emissions in OECD. Appl. Energy 2018, 231, 937–950. 10.1016/j.apenergy.2018.09.179.

[ref48] Scherer L.; de Koning A.; Tukker A. BRIC and MINT countries’ environmental impacts rising despite alleviative consumption patterns. Sci. Total Environ. 2019, 665, 52–60. 10.1016/j.scitotenv.2019.02.103. 30772578

[ref49] Ang B.; Zhang F.; Choi K. Factorizing changes in energy and environmental indicators through decomposition. Energy 1998, 23, 489–495. 10.1016/S0360-5442(98)00016-4.

[ref50] Tang X.; Jin Y.; McLellan B. C.; Wang J.; Li S. China’s coal consumption declining—Impermanent or permanent?. Resour. Conserv. Recycl. 2018, 129, 307–313. 10.1016/j.resconrec.2016.07.018.

[ref51] Ang B. W. LMDI decomposition approach: A guide for implementation. Energy Policy 2015, 86, 233–238. 10.1016/j.enpol.2015.07.007.

[ref52] Editorial Board of China Power Yearbook. China Electric Power Yearbook, 2008–2018.

[ref53] China Electricity Council. Materials of National Energy Efficiency Benchmarking Competition for Thermal Power Units, 2018.

[ref54] Ding X.; Tian W.; Chen Q.; Wei G. Policies on water resources assessment of coastal nuclear power plants in China. Energy Policy 2019, 128, 170–178. 10.1016/j.enpol.2019.01.008.

[ref55] Ministry of Water Resources of the People’s Republic of China. Water Resources Bulletin of China in 2020, 2021.

[ref56] China Electricity Council. Annual Compilation of Statistics of Power Industry, 2018.

[ref57] Zhang C.; Zhong L.; Liang S.; Sanders K. T.; Wang J.; Xu M. Virtual scarce water embodied in inter-provincial electricity transmission in China. Appl. Energy 2017, 187, 438–448. 10.1016/j.apenergy.2016.11.052.

[ref58] Qu S.; Liang S.; Xu M. CO2 Emissions Embodied in Interprovincial Electricity Transmissions in China. Environ. Sci. Technol. 2017, 51, 10893–10902. 10.1021/acs.est.7b01814. 28792748

[ref59] China Electricity Council. Annual Development Report of China’s Power Industry, 2020.

[ref60] Ministry of Water Resources of the People’s Republic of China. Lengths of Major Rivers in China, 2003.

[ref61] Ministry of Water Resources of the People’s Republic of China. Bulletin of River Sediment in China, 2020.

[ref62] Chinese Hydraulic Engineering Society. China Water Resources, 2016.

[ref63] Ministry of Water Resources of the People’s Republic of China. China Water Statistical Yearbook, 2018.

[ref64] International Energy Agency. World Energy Outlook 2020, 2020.

[ref65] Standardization Administration. Norm of Water Intake Part 1: Electric Power Production, 2012.

[ref66] National Energy Administration. Water Saving Guideline for Thermal Power Plant, 2018.

[ref67] Pan L.; Liu Z.; Zhang B. Comprehensive analysis and related measures on current situation ofwater saving of thermal power generation in China. Electr. Power 2017, 50, 158–163.

[ref68] Xu H.; Lei Y.; Lu C.; Xie Y. Comparative analysis of direct and indirect air cooling system for thermal power unit selection. Air Pollut. Control 2014, 101–104.

[ref69] Kuipers K. J. J.; Hellweg S.; Verones F. Potential Consequences of Regional Species Loss for Global Species Richness: A Quantitative Approach for Estimating Global Extinction Probabilities. Environ. Sci. Technol. 2019, 53, 4728–4738. 10.1021/acs.est.8b06173. 30995027

[ref70] Byers E. A.; Hall J. W.; Amezaga J. M. Electricity generation and cooling water use: UK pathways to 2050. Global Environ. Change 2014, 25, 16–30. 10.1016/j.gloenvcha.2014.01.005.

[ref71] Rosa L.; Reimer J. A.; Went M. S.; D’Odorico P. Hydrological limits to carbon capture and storage. Nat. Sustainability 2020, 3, 658–666. 10.1038/s41893-020-0532-7.

[ref72] Kati V.; Kassara C.; Vrontisi Z.; Moustakas A. The biodiversity-wind energy-land use nexus in a global biodiversity hotspot. Sci. Total Environ. 2021, 768, 144471 10.1016/j.scitotenv.2020.144471. 33454485

[ref73] Wu X.; Shao L.; Chen G.; Han M.; Chi Y.; Yang Q.; Alhodaly M.; Wakeel M. Unveiling land footprint of solar power: A pilot solar tower project in China. J. Environ. Manage. 2021, 280, 111741 10.1016/j.jenvman.2020.111741. 33352380

[ref74] Barbarossa V.; Schmitt R. J. P.; Huijbregts M. A. J.; Zarfl C.; King H.; Schipper A. M. Impacts of current and future large dams on the geographic range connectivity of freshwater fish worldwide. Proc. Natl. Acad. Sci. U.S.A. 2020, 117, 3648–3655. 10.1073/pnas.1912776117. 32015125 PMC7035475

[ref75] Heino J.; Paavola R.; Virtanen R.; Muotka T. Searching for biodiversity indicators in running waters: do bryophytes, macroinvertebrates, and fish show congruent diversity patterns?. Biodivers. Conserv. 2005, 14, 415–428. 10.1007/s10531-004-6064-z.

[ref76] Tendall D. M.; Hellweg S.; Pfister S.; Huijbregts M. A. J.; Gaillard G. Impacts of River Water Consumption on Aquatic Biodiversity in Life Cycle Assessment—A Proposed Method, and a Case Study for Europe. Environ. Sci. Technol. 2014, 48, 3236–3244. 10.1021/es4048686. 24506171

[ref77] Sun Z.; Zhang L.; Duan Z. Number and scale of hydropower station projects in China. China Water Resour. 2013, 12–13.

