# Supplementary material for: Biodiversity Loss from Freshwater Use for China’s Electricity Generation
Source: Environ Sci Technol. 2022 Feb 18;56(5):3277–87. doi: 10.1021/acs.est.1c07155 (PMC8892834; doi:10.1021/acs.est.1c07155)
Supplement: Supplementary file 1 — es1c07155_si_001.pdf [file es1c07155_si_001.pdf]

Supplementary information of

Biodiversity loss from freshwater use for China's electricity generation

Yi Jin,[*,†] Paul Behrens,[†,‡] Arnold Tukker,[†,§] Laura Scherer[†]

[†]Institute of Environmental Sciences (CML), Leiden University, 2333 CC Leiden, The Netherlands

[‡]Leiden University College The Hague, Leiden University, 2595 DG The Hague, The Netherlands

[§]Netherlands Organization for Applied Scientific Research TNO, 2595 DA The Hague, The Netherlands

Number of pages: 14

Number of figures: 5

Number of tables: 3

S1 Estimates of water consumption

We assessed the provincial water consumption factors for thermal power and hydropower generation using the method in Jin et al. [1]. Based on the factors, the water consumption of electricity generation in each year is calculated as follows:

$$WC = \sum_i WC_i = \sum_i (TWC_i + HWC_i) = \sum_i (TF_i \cdot TP_i + HF_i \cdot HP_i) \qquad (1)$$

In which, WC gives the national water consumption for electricity generation (m^3); WC_i the water consumption for electricity generation in provinces i (m^3); TWC_i the water consumption for thermal power generation in province i (m^3); HWC_i the water consumption for hydropower generation in province i (m^3); TF_i the water consumption factor for thermal power generation in province i (m^3/GWh); HF_i the water consumption factor for hydropower generation in province i (m^3/GWh); TP_i the thermal power generation in province i (GWh); HP_i the hydropower generation in province i (GWh).

S2 Characterization factors for water consumption

Water consumed for electricity generation is not returned to the river. The influence of reduced flow rates on aquatic biodiversity can be quantified with the global species-discharge model, an index of habitat space, feeding and reproductive opportunities. This model is developed based on native fish species and river discharges in various river basins [2]. This model assumes a positive correlation between the number of freshwater fish species and average river discharges at the mouth of river basins.

$$R = 4.2 \cdot Q_{mouth,i}^{0.4} \qquad (2)$$

where R is the freshwater fish species richness and $Q_{mouth,i}$ is the annual average river discharge at the river mouth of basin i (m^3/s).

The species-discharge relationship can be used to calculate characterization factors for water consumption that specify freshwater fish species loss per unit of reduced river discharge for river basins in different regions. Characterization factors (CF_c) for

water consumption reflect the impact of water use due to human activities on freshwater biodiversity loss.

$$CF_{c,i} = FF_i \cdot EF_i = \frac{dQ_{mouth,i}}{dW_i} \cdot \left(\frac{dPDF_i}{dQ_{mouth,i}} \cdot V_i\right) \quad (3)$$

where FF_i is the fate factor of river basin i, EF_i is the effect factor of river basin i (PDF·m^3·yr·m^{-3}), $dQ_{mouth,i}$ is the marginal change in water discharge at the river mouth in basin i (m^3·yr^{-1}), dW_i is the marginal change in water consumption by human activities in river basin i (m^3·yr^{-1}), $dPDF_i$ is the marginal change in the potentially disappeared fraction of the freshwater fish species due to the marginal river discharge change $dQ_{mouth,i}$ and V_i is the volume of river basin i (m^3). The $dQ_{mouth,i}/dW_i$ is assumed to be equal to one, indicating that a change in water consumption is fully reflected in a change in water discharge at the mouth for that river basin.

$$\frac{dPDF_i}{dQ_{mouth,i}} = \frac{dR_i}{R_i \cdot dQ_{mouth,i}} = \frac{4.2 \cdot 0.4 \cdot Q_{mouth,i}^{0.4-1}}{4.2 \cdot Q_{mouth,i}^{0.4}} = \frac{0.4}{Q_{mouth,i}} \quad (4)$$

The river volumes (m^3) for all river basins are calculated according to Hanafiah et al. [3] as follows:

$$V_i = 0.47 \cdot \left(\frac{Q_{mouth,i}}{2}\right)^{0.9} \cdot L_i \quad (5)$$

where V_i is the water volume in river basin i (m^3), $Q_{mouth,i}$ is the discharge at the river mouth in basin i (m^3·yr^{-1}), Li is the length of river i (m).

China can be divided into the following river basins: Huaihe, Haihe, Yellow, Yangtze, Pearl, Southeast, Southwest, Continental, Songhua and Liaohe river basins. The characterization factors are calculated for these river basins. Specifically, Qiantang and Min rivers are the representatives of the Southeast river basin. The characterization factors of Qiantang and Min river basins are calculated for Zhejiang and Fujian provinces since they are the largest river basins of the two provinces, respectively [4, 5]. Talimu is the largest river in the Continental basin, and its characterization factor is calculated for this basin. In terms of Southwest, Brahmaputra is the largest river basin of Tibet, and its characterization factor is calculated for Tibet [6]. Nandu river is the largest river of Hainan province, and its

characterization factor is calculated for Hainan. The discharges at the river mouth and the river length are obtained from the Ministry of Water Resources [4, 7-9].

S3 Thermal pollution of power production

S3.1 Thermal pollution from thermal power

In power plants with once-through cooling systems, water from a freshwater body is used to absorb heat from the working fluid in the condenser. The entire volume of heated water is then discharged back into the water body. In the Rankine cycle of steam-electric generating units, pumps and boilers add heat to liquid water, which is converted into steam during that process. The high-pressure steam then expands in the turbine producing power. Upon exiting the turbine, the steam passes through the condenser where heat is rejected from the system turning the working fluid into a saturated liquid, ready to re-enter the pump. To calculate the rate of heat rejected in each cycle, the difference in enthalpy of the working fluid on either side of the condenser must be multiplied by the steam flow rate. The thermal pollution to water bodies from thermal power is calculated using the method of Raptis and Pfister [10]. The heat rejection rates of thermal power are assessed as follows:

$$Q_t = LF \cdot m_{steam}(h_b - h_a)/1000 \quad (6)$$

Where Q is the heat rejection rate (MW), LF is the load or capacity factor of electricity generating units, which are derived from Jin et al. [11], m_{steam} is the steam flow rate at the high-pressure turbine (kg/s), h_b-h_a is the difference in enthalpy of the working fluid on either side of the condenser (kJ/kg).

The steam flow rate can be calculated as:

$$m_{steam} = \beta \cdot C_{gross} \quad (7)$$

Where C_{gross} is the gross generating capacity (MW) and β is a constant (0.830 kg s^{-1} MW^{-1} [10]).

Reheat cycles can be added into the Rankine cycle, increasing the generation efficiency and thus reducing fuel inputs. When reheat cycles are employed, the steam passes first through a high-pressure turbine and, after being reheated, through a low-

pressure turbine. 94% of China's units with an installed capacity of 100-220 MW use a reheat system, whereas all 300-1000 MW units use a reheat system [12]. For a reheat system, the ratio (r) of the steam flow at the entry of low-pressure turbine to the steam flow at the entry of high-pressure turbine is inserted to scale the rejection rate:

$$Q_t = LF \cdot m_{steam} \cdot r \cdot (h_b - h_a)/1000 \quad (8)$$

Where r=0.85 is used for China's units in this study, referring to Yan et al. [13] and Cheng et al. [14]. h_a is related to the water temperature withdrawn for use in the condenser [10]. An additional necessary piece of information for all thermodynamic cycles is the temperature of the freshwater withdrawn for use in the condenser. To obtain these values, the georeferenced power plants are overlaid onto gridded estimates (at 10 km spatial resolution) of water temperatures [15]. The average over 15 years (2000-2014) is used to minimize the impacts of very warm or very cold years on the water temperature estimates. For every generating unit then, mean monthly naturalized water temperatures are extracted. The information on power plants is sourced from the China Electricity Council [12], Global Coal Plant Tracker [16], World Electric Power Plants Database [17] and our previous study [11]. The results of plant-level thermal pollution and its impacts are then aggregated to the provincial level.

S3.2 Thermal pollution from hydropower

Hydropower also produces heat during operation, though its thermal pollution is smaller than that of thermal power with a once-through cooling system because of its higher energy efficiency. The thermal emission of hydropower can be calculated as follows:

$$Q_h = LF \cdot C_{gross} \cdot HR \quad (9)$$

Where HR is the heat emission rate of China's hydropower, referring to Xu et al. [18] and Yan and Hao [19]; here, HR is 1.8%. The definitions of LF and C_{gross} are the same as those in Equations 7-9.

There are approximately 47,000 hydropower stations in China [20]. It is infeasible to assess the thermal emission and biodiversity impacts at the plant level because of data limitations. We made assessments at the provincial level by changing equation 10 to

11:

$$Q_h = LF_p \cdot C_p \cdot HR \quad (10)$$

Where LF_p is the provincial load or capacity factor of hydropower, C_p is the provincial installed capacity of hydropower (MW). The values of LF_p and C_p are obtained from the National Bureau of Statistics [21].

S4 Biodiversity impacts assessments

Biodiversity loss caused by freshwater consumption: Electricity generation can cause aquatic biodiversity loss because of its water use [22, 23]. Surface water consumption impacts aquatic biodiversity. Water consumption is translated to impacts on aquatic biodiversity by characterization factors expressed as a potentially disappeared fraction of species (unit: PDF m^3yr /m^3) [3].

$$WBL_i = WC_i \cdot CF_{c,i} \quad (11)$$

Where WBL_i gives the biodiversity loss caused by water consumption for electricity generation in province i (PDF m^3 yr); WC_i the water consumption for electricity generation in province i (m^3); $CF_{c,i}$ the biodiversity loss per unit of water consumption for electricity generation in province i (PDF m^3 yr/m^3);

Biodiversity loss caused by thermal emissions: The factor of local biodiversity impacts from thermal emissions is obtained from Raptis et al. [23] .

The biodiversity loss caused by electricity generation is calculated as follows:

$$TBL_i = TBLT_i + TBLH_i = \sum_n Q_{t,n,i} \cdot TBF_{n,i} + Q_{h,i} \cdot PTBF_i \quad (12)$$

Where TBL_i gives the biodiversity loss caused by thermal emissions of electricity generation in province i (PDF m^3 yr); $TBLT_i$ the biodiversity loss caused by thermal emissions from thermal power in province i (PDF m^3 yr); $TBLH_i$ the biodiversity loss caused by thermal emissions from hydropower in province i (PDF m^3 yr); $Q_{t,n,i}$ the thermal emissions from the thermal power plant n in province i (MJ); $TBF_{n,i}$ the biodiversity loss per unit of thermal emissions at the location of plant n in province i (PDF m^3 yr/MJ); n the thermal power plants with once-through cooling systems in

province i; $Q_{h,i}$ the thermal emissions from hydropower in province i (MJ); $PTBF_i$ the biodiversity loss per unit of thermal emissions in province i (PDF m^3 yr/MJ); the characterization factors are derived from Raptis et al. [23], where global gridded freshwater thermal pollution CFs are assessed. We extract the gridded CFs for thermal power and China's CFs for hydropower at monthly resolution.

S5 Estimates of water stress

The Water Stress Index is calculated according to Pfister et al. [24], which is adapted from the water withdrawal-to-availability indicator by applying a logistic function to acquire continuous values between 0.01 and 1. The equation is as follows:

$$WSI = \frac{1}{1 + e^{-6.4 \cdot WTA^*}\left(\frac{1}{0.01} - 1\right)} \quad (13)$$

Where WSI is the water stress index. WTA^* is a modified WTA indicator considering the difference for watersheds with and without strongly regulated flows. Four levels of water stress are classified in the WSI, i.e. minor (0.01-0.09); moderate (0.09-0.5); severe (0.5-0.91); and extreme (0.91-1). The water stress indexes are calculated for 2017 based on the water withdrawal and availability from the Ministry of Water Resources [25].

S6 Converting local impacts to global impacts

Kuipers et al. estimated global extinction probabilities (GEPs) based on species range sizes, species vulnerabilities, and species richness, indicating to what extent regional species loss in the respective area may contribute to global species loss. They generate them for marine, terrestrial, and freshwater species groups on the local (i.e., 0.05° × 0.05° grid) and ecoregion scale [26]. The regional fractions of freshwater species losses are then multiplied with the corresponding GEPs to calculate potential global fractions of extinctions:

$$GBL_i = BL_i/V_i \cdot GEP_i \quad (14)$$

Where GBL_i gives the potential global biodiversity loss in province i (PDF yr); V_i is

the volume of the representative river in province *i*; GEP_i is the global extinction probability in province *i*, calculated by aggregating the cell-level GEPs from Kuipers et al. [26] within province *i*.

Supplementary figures

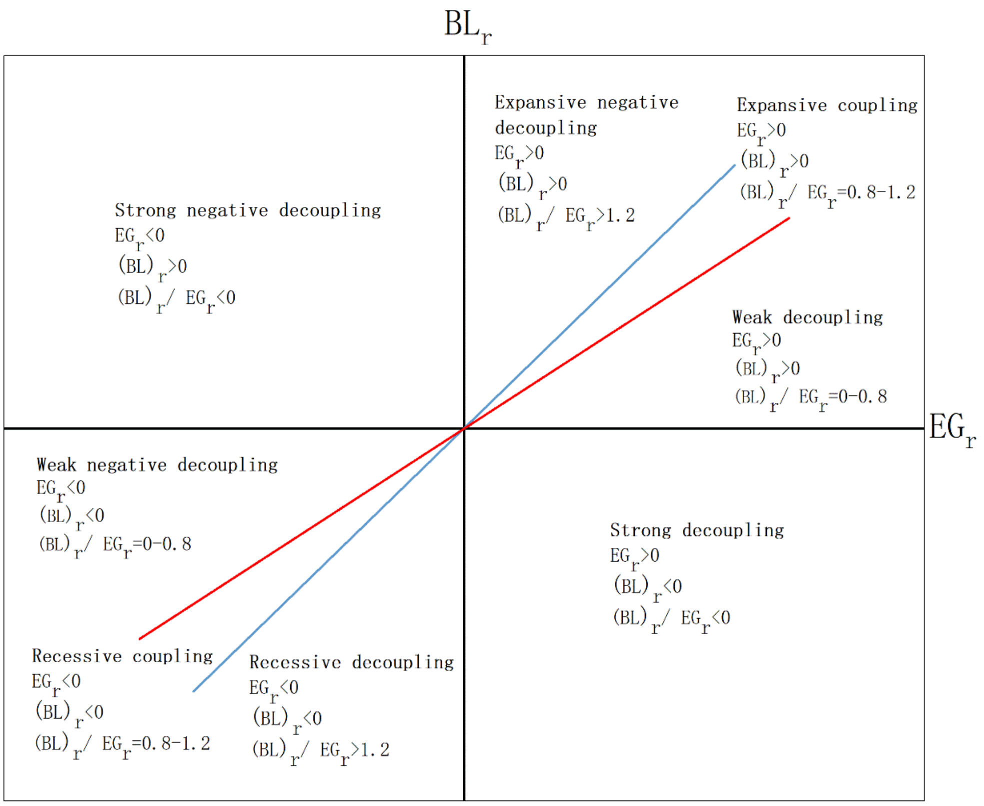

Supplementary Figure S1. The decoupling state quadrant map corresponding to the decoupling degree. Here, BL_r=$\Delta BL/BL_{t-1}$, EG_r=$\Delta EG/EG_{t-1}$. This map is modified from Tapio [27].

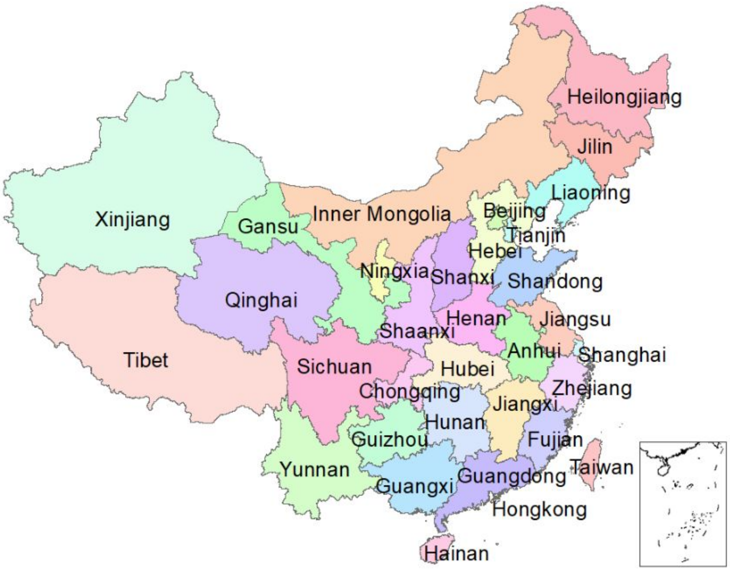

Supplementary Figure S2. China's provinces.

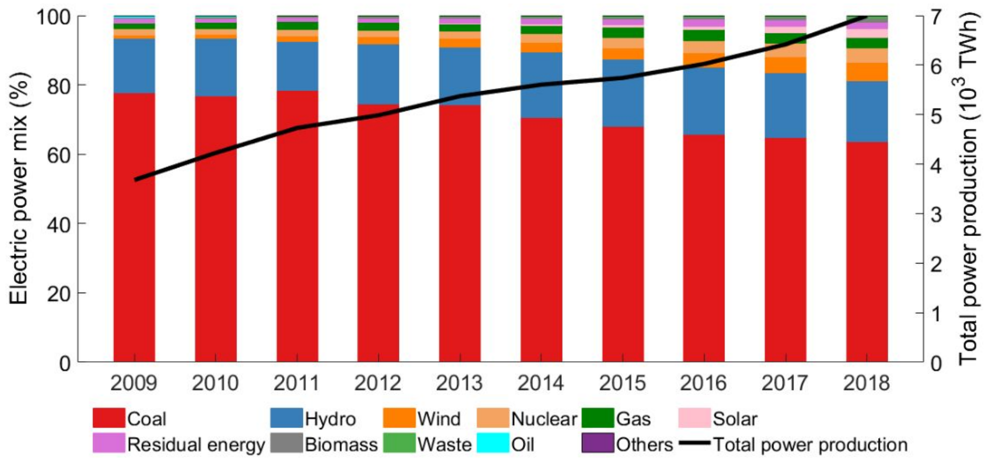

Supplementary Figure S3. The electric power mix in China.

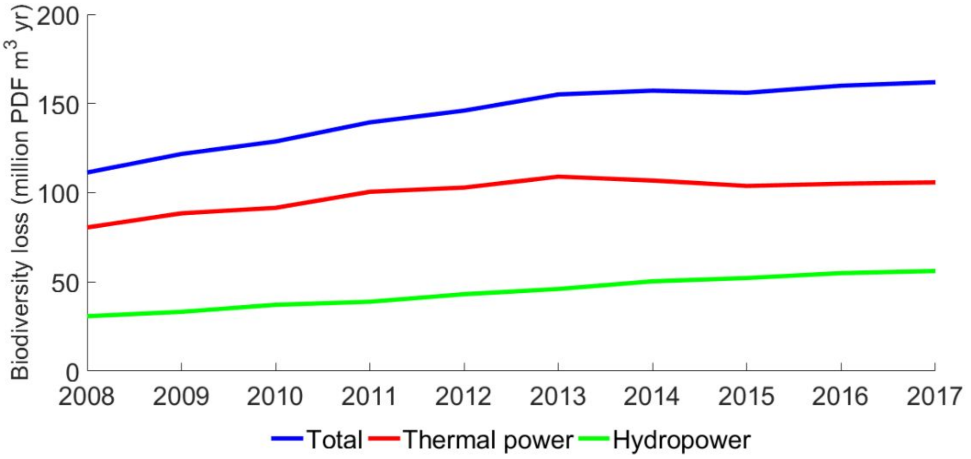

Supplementary Figure S4. The biodiversity loss by freshwater use for China's electricity generation during 2008-2017.

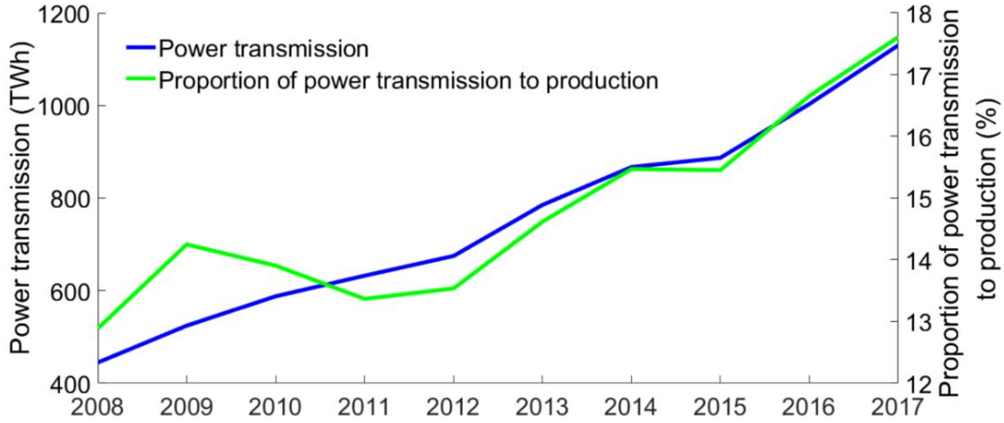

Supplementary Figure S5. The total amount of interprovincial power transmission in China.

Supplementary tables

Supplementary Table S1. The provincial characterization factors of water consumption impacts on local biodiversity.

**Province**	**Characterization factor ($PDF \cdot m^3 \cdot yr \cdot m^{-3}$)**	**Province**	**Characterization factor ($PDF \cdot m^3 \cdot yr \cdot m^{-3}$)**
Beijing	1.76E-03	Hubei	7.19E-03
Tianjin	1.76E-03	Hunan	7.19E-03
Hebei	1.76E-03	Guangdong	2.78E-03
Shanxi	8.17E-03	Guangxi	2.78E-03
InnerMongolia	8.17E-03	Hainan	5.79E-04
Liaoning	2.51E-03	Chongqing	7.19E-03
Jilin	2.84E-03	Sichuan	7.19E-03
Heilongjiang	2.84E-03	Guizhou	7.19E-03
Shanghai	7.19E-03	Yunnan	3.99E-03
Jiangsu	7.19E-03	Xizang	2.79E-03
Zhejiang	6.76E-04	Shaanxi	8.17E-03
Anhui	7.19E-03	Gansu	8.17E-03
Fujian	8.14E-04	Qinghai	8.17E-03
Jiangxi	7.19E-03	Ningxia	8.17E-03
Shandong	1.50E-03	Xinjiang	3.34E-03
Henan	1.50E-03		

Supplementary Table S2. The provinces' full names and abbreviations used in Figure 4.

**Full name**	**Abbreviation**	**Full name**	**Abbreviation**
Anhui	AH	Liaoning	LN
Beijing	BJ	Inner Mongolia	NM
Fujian	FJ	Ningxia	NX
Gansu	GS	Qinghai	QH
Guangdong	GD	Shandong	SD
Guangxi	GX	Shanxi	SX
Guizhou	GZ	Shaanxi	SN
Hainan	HI	Shanghai	SH
Hebei	HE	Sichuan	SC
Henan	HA	Tianjin	TJ
Heilongjiang	HL	Xizang	XZ
Hubei	HB	Xinjiang	XJ
Hunan	HN	Yunnan	YN
Jilin	JL	Zhejiang	ZJ
Jiangsu	JS	Chongqing	CQ
Jiangxi	JX		

Supplementary Table S3. The decoupling degree and decoupling state (see Supplementary Figure 1) between biodiversity loss and electricity generation.

Time period	Decoupling degree	Decoupling state
2008-2009	1.4	Expansive negative decoupling
2009-2010	0.39	Weak decoupling
2010-2011	0.7	Weak decoupling
2011-2012	0.87	Expansive coupling
2012-2013	0.8	Expansive coupling
2013-2014	0.31	Weak decoupling
2014-2015	-0.31	Strong decoupling
2015-2016	0.52	Weak decoupling
2016-2017	0.19	Weak decoupling

Supplementary references

(1) Jin, Y., Behrens, P., Tukker, A., Scherer, L. Water use of electricity technologies: A global meta-analysis. Renew Sustain Energy Rev. 2019,115,109391.

(2) Xenopoulos, M.A., Lodge, D.M., Alcamo, J., Märker, M., Schulze, K., Van Vuuren, D.P. Scenarios of freshwater fish extinctions from climate change and water withdrawal. Global Change Biol. 2005,11,1557-64.

(3) Hanafiah, M.M., Xenopoulos, M.A., Pfister, S., Leuven, R.S.E.W., Huijbregts, M.A.J. Characterization Factors for Water Consumption and Greenhouse Gas Emissions Based on Freshwater Fish Species Extinction. Environ Sci Technol. 2011,45,5272-8.

(4) Ministry of Water Resources of the People's Republic of China. Bulletin of river sediment in China. 2020.

(5) Committee of Yearbook of China water resources. Yearbook of China water resources. 2014.

(6) Tibet academy of agricultural and animal husbandry sciences. Water resources of Tibet. 2020.

(7) Ministry of Water Resources of the People's Republic of China. Lengths of major rivers in China. 2003.

(8) Chinese Hydraulic Engineering Society. China Water Resources. 2016.

(9) Ministry of Water Resources of the People's Republic of China. China Water Statistical Yearbook. 2018.

(10) Raptis, C.E., Pfister, S. Global freshwater thermal emissions from steam-electric power plants with once-through cooling systems. Energy. 2016,97,46-57.

(11) Jin, Y., Behrens, P., Tukker, A., Scherer, L. The energy-water nexus of China's interprovincial and seasonal electric power transmission. Appl Energy. 2021,286,116493.

(12) China Electricity Council. Materials of national energy efficiency benchmarking competition for thermal power units. 2018.

(13) Yan, S., Zeng, D., Liu, J., Wang, W., Xie, X. A Soft-sensor Method of Reheat Steam Flow Based on Simplified Heat-balance Equation. Chin Soc for Elec Eng. 2011,31,114-9.

(14) Cheng, F., Fan, S., Zhao, J. Improve Thermal Efficiency by Stabilizing Steam Parameters of Power Plant Boiler. Energy and Energy Conversion. 2013,71-4.

(15) Wanders, N., van Vliet, M.T.H., Wada, Y., Bierkens, M.F.P., van Beek, L.P.H.R. Global monthly water temperature dataset, derived from dynamical 1-D water-energy routing model (DynWat) at 10 km spatial resolution (1.1). 2018.

(16) CoalSwarm. Global Coal Plant Tracker. 2019.

(17) Platts Energy InfoStore Utility Data Institute. World Electric Power Plants Database. 2018.

(18) Wei, X., Xia Binqiang, Yuzhu, W. Measuring Method of Hydroelectric Generator Efficiency Based on Infrared Thermal Imaging Technology. China Water Power & Electrification. 2018,6,12-8.

(19) Yan, Y., Hao, J. Consumption measurement and efficiency calculation for large-sized hydraulic generators based on calorimetric method. Hunnan Electric Power. 2014,34,8-11.

(20) Sun, Z., Zhang, L., Duan, Z. Number and scale of hydropower station projects in China. China Water Resources. 2013,12-3.

(21) National Bureau of Statistics of China. Regional power production. 2019.

(22) Pfister, S., Suh, S. Environmental impacts of thermal emissions to freshwater: spatially explicit fate and effect modeling for life cycle assessment and water footprinting. The International Journal of

Life Cycle Assessment. 2015,20,927-36.
(23) Raptis, C.E., Boucher, J.M., Pfister, S. Assessing the environmental impacts of freshwater thermal pollution from global power generation in LCA. Sci Total Environ. 2017,580,1014-26.
(24) Pfister, S., Koehler, A., Hellweg, S. Assessing the Environmental Impacts of Freshwater Consumption in LCA. Environ Sci Technol. 2009,43,4098.
(25) Ministry of Water Resources of the People's Republic of China. Water Resources Bulletin of China in 2017. 2018.
(26) Kuipers, K.J.J., Hellweg, S., Verones, F. Potential Consequences of Regional Species Loss for Global Species Richness: A Quantitative Approach for Estimating Global Extinction Probabilities. Environ Sci Technol. 2019,53,4728-38.
(27) Tapio, P. Towards a theory of decoupling: degrees of decoupling in the EU and the case of road traffic in Finland between 1970 and 2001. Transport Policy. 2005,12,137-51.
